# Responsible artificial intelligence in public health: a Delphi study on risk communication, community engagement and infodemic management

**DOI:** 10.1136/bmjgh-2024-018545

**Published:** 2025-05-23

**Authors:** Daniela Mahl, Mike S Schäfer, Stefan Adrian Voinea, Keyrellous Adib, Ben Duncan, Cristiana Salvi, David Novillo-Ortiz

**Affiliations:** 1 Department of Communication and Media Research, University of Zurich, Zurich, Switzerland; 2 World Health Organization Regional Office for Europe, Copenhagen, Denmark

**Keywords:** Global Health, Public Health, Interdisciplinary Research, Qualitative study, Descriptive study

## Abstract

**Introduction:**

Artificial intelligence (AI) holds the potential to fundamentally transform how public health authorities use risk communication, community engagement and infodemic management (RCCE-IM) to prepare for, manage and mitigate public health emergencies. As research on this crucial transformation remains limited, we conducted a modified Delphi study on the impact of AI on RCCE-IM.

**Methods:**

In two successive surveys, 54 experts―scholars with expertise in public health, digital health, health communication, risk communication and AI, as well as RCCE-IM professionals―from 27 countries assessed opportunities, challenges and risks of AI, anticipated future scenarios, and identified principles and actions to facilitate the responsible use of AI. The first Delphi round followed an open, exploratory approach, while the second sought to prioritise and rank key findings from the initial phase. Qualitative thematic analysis and statistical methods were applied to evaluate responses.

**Results:**

According to the expert panel, AI could be highly beneficial, particularly for risk communication (eg, tailoring messages) and infodemic management (eg, social listening), while its utility for fostering community engagement was viewed more critically. Challenges and risks affect all three components of RCCE-IM equally, with algorithmic bias and privacy breaches being of particular concern. Panellists anticipated both optimistic (eg, democratisation of information) and pessimistic (eg, erosion of public trust) future scenarios. They identified seven principles for the responsible use of AI for public health practices, with equity and transparency being the most important. Prioritised actions ranged from regulatory measures, resource allocation and feedback loops to capacity building, public trust initiatives and educational training.

**Conclusion:**

To responsibly navigate the multifaceted opportunities, challenges and risks of AI for RCCE-IM in public health emergencies, clear guiding principles, ongoing critical evaluation and training as well as societal collaboration across countries are needed.

**WHAT IS ALREADY KNOWN ON THIS TOPIC**

- Artificial intelligence (AI) is poised to fundamentally transform several health domains, including public health, medicine, clinical care, medical research and education.
- Despite the well-recognised importance of public health authorities’ risk communication, community engagement and infodemic management (RCCE-IM) in responding to health crises throughout the emergency cycle, little scholarly attention has been paid to AI’s transformative potential in this critical area.

**WHAT THIS STUDY ADDS**

- To the best of our knowledge, this Delphi study represents the first international, multidisciplinary and transdisciplinary effort to comprehensively assess how AI impacts RCCE-IM interventions. Our findings demonstrate AI’s potential to profoundly reshape RCCE-IM, while also emphasising the challenge of responsibly leveraging these opportunities amidst substantial risks, underscoring AI’s double-edged nature.
- The transformative future scenarios envisioned by the expert panel highlight the need for public health authorities to proactively harness AI’s potential to democratise information, while mitigating the risk that the use of AI will lead to an erosion of trust in public authorities.

**HOW THIS STUDY MIGHT AFFECT RESEARCH, PRACTICE OR POLICY**

- This exploratory assessment of the responsible development, implementation and regulation of AI for RCCE-IM provides evidence-based and actionable guidance for scholars, RCCE-IM professionals and policymakers to collaboratively identify areas of consensus and develop policies that are globally relevant yet sensitive to specific cultures, regions, organisations and technologies.

## Introduction

 Public health emergencies of the 21st century―from disease outbreaks like the COVID-19 pandemic and mpox to humanitarian crises such as the war in Ukraine and the Israeli-Palestinian conflict―have demonstrated a critical lesson: managing and mitigating such emergencies depends not only on the availability of health services and interventions but also on their acceptance and use by affected populations.^1^ Risk communication, community engagement and infodemic management (RCCE-IM) play a critical role in promoting adherence to health recommendations^2^ and ensuring the effectiveness of emergency responses at all stages, from prevention and preparedness to response and recovery.^3 4^

Each component of RCCE-IM is dedicated to distinct interventions. According to the WHO,^2 5^ *risk communication* provides accurate, timely and relevant information, tailored to the population’s perceptions and health needs, enabling those at risk to make informed decisions. *Community engagement* actively involves affected communities in designing and implementing interventions, promoting positive health outcomes and cultural sensitivity in emergencies and beyond. *Infodemic management* entails the continuous monitoring of health narratives to detect inaccuracies, outdated information and information voids, ensuring that accurate information guides public behaviour. The significance of RCCE-IM in public health emergencies is widely recognised in international frameworks, including the legally binding International Health Regulations and WHO’s more recent global and regional frameworks Health Emergency Preparedness, Response, and Resilience and Preparedness V.2.0. These frameworks place the responsibility for developing, implementing and evaluating RCCE-IM strategies on public health authorities in collaboration with whole-of-government and whole-of-society stakeholders.

Global health emergencies like the COVID-19 pandemic, as well as technological advances, can be powerful drivers of transformation,^6 7^ prompting public health authorities to advance or adapt RCCE-IM strategies. One of these transformative technologies is artificial intelligence (AI), which refers to machine-based systems that use input data ‘to generate outputs such as predictions, content, recommendations or decisions’.^8^ A substantial body of academic research and impact assessments^9^,^13^ suggests that AI is poised to fundamentally reshape several health domains, including public health,^14^ medicine,^15^ clinical care,^16^ medical research^17^ and education.^18^ To date, AI has been implemented in various healthcare fields, including cardiology,^19^ pharmacology^20^ and radiology,^21^ with proposed promises such as assisting in mammography interpretation,^22^ generating discharge summaries,^23^ advancing drug development^24^ and utilising AI-equipped robots as simulated patients.^25^ Despite AI’s potential benefits for public health, it also presents challenges and risks, including the generation of inaccurate content with the risk of hallucinations,^26^ misrepresentation of medical evidence,^27^ poor model interpretability,^28^ perpetuation of bias^29^ and insufficient infrastructure.^30^

Notably, the current discussion of the promises and perils of AI for public health remains disproportionately oriented towards clinical care, education and research, while comparatively little is known about public health practices^31 32^—especially in the context of health emergencies and how to prepare for, manage and mitigate such crises. To address this critical shortcoming, we conducted a modified two-wave Delphi study involving a multidisciplinary and transdisciplinary expert panel of 54 scholars and RCCE-IM professionals from 27 countries to assess the opportunities, challenges and risks associated with AI, anticipate future scenarios and identify key principles and actions to facilitate the responsible use of AI for RCCE-IM.

## Methods

### The Delphi method

The Delphi method is a highly regarded approach for ‘expert elicitation and stakeholder engagement’^33^ to assess current knowledge,^34^ identify areas of consensus,^35^ develop guidelines,^36^ prioritise actions^37^ or anticipate future scenarios.^38^ It is particularly valuable in rapidly evolving or complex areas where empirical evidence is inconclusive or scarce.^39^ As the role of AI in managing public health emergencies remains largely unexplored while technological advances are rapidly evolving, this study aims to map the current state of knowledge, anticipate future scenarios and identify priorities for action. Such an initial exploratory assessment is crucial, as it provides an evidence-based foundation for future research to identify areas of consensus and enables public health organisations to develop context-sensitive guidelines and implementation frameworks.

The fundamental premise of Delphi studies is the progressive aggregation of anonymous expert assessments through surveys or interviews, with each iteration building on the collective insights of the preceding one.^40^ The iterative process allows participants to refine their views based on the perspectives of others,^41^ while the anonymity ensures that all experts are given equal weight, minimising potential biases arising from dominant individuals.^42^

In the health sciences, the Delphi method has been applied extensively, for instance, to develop guidelines for epidemiologic practice,^43^ global COVID-19 vaccination strategies^44^ and long COVID management.^45^ It has also been used to examine the role of AI in emergency medicine,^46^ mental health,^47^ primary care^48^ and surgery.^41^

### The Delphi design and analysis

In accordance with this Delphi study’s objectives, the study design was adapted. In contrast to traditional consensus-oriented Delphi studies, in which the number of rounds is determined by predefined agreement thresholds or response stability criteria,^49^ we opted for an open and exploratory two-wave approach, provided that no expert objected to the results of the final wave—a condition that was met in this study.

Accordingly, two online surveys were conducted between May and July 2024 (see online supplemental appendix A for both questionnaires). The first round adopted an exploratory approach, with the questionnaire consisting of open-ended questions covering five domains: (1) AI opportunities, (2) AI challenges and risks, (3) future AI scenarios, (4) principles and (5) required actions for responsible AI use. Verbatim responses were systematically reviewed, with similar items consolidated and rephrased for clarity while preserving the original meaning. Subsequently, responses were analysed using qualitative thematic analysis^50^ in MAXQDA, a software for computer-assisted qualitative data analysis.

The second round was designed to prioritise and rank the key findings from the initial phase. Accordingly, experts were asked to identify the three most important opportunities, challenges, risks, principles and required actions. Rankings were analysed through weighted statistical analysis performed in R. The rank of each item was determined by the number of experts who identified it as the most important (Top1, weighted by a factor of 3), second most important (Top2, weighted by a factor of 2) and third most important (Top3). For future scenarios, experts rated the likelihood of each scenario occurring in the next 5 years on a 5-point Likert scale (1=‘not at all likely’ to 5=‘very likely’) and assessed the importance of harnessing or mitigating these scenarios in the next 5 years (1=‘not at all important’ to 5=‘very important’). In the second wave, the experts were also invited to provide feedback on the results of the previous wave, encouraging them to disagree with their peers’ assessment or to refine their own. The design and analysis of this Delphi study, conducted in accordance with the ethics guidelines of the University of Zurich and the WHO, were informed by regular consultations with an advisory group of RCCE-IM and AI experts from the WHO Regional Office for Europe.

### The Delphi expert panel

To assemble an international, multidisciplinary and transdisciplinary expert panel, we employed a multistage sampling strategy, using purposive snowball sampling^51^ and maximum variation sampling.^52^ Based on a literature analysis and the authors’ networks, two categories of experts were identified: (1) scholars with recognised expertise in public health, digital health, health communication, risk communication, AI or related fields and (2) RCCE-IM professionals.

While criteria for defining expertise vary,^38^ we used a combination of the following: senior or specialist positions in academia or public health authorities; at least 3 years of professional experience; authorship of peer-reviewed publications in high-impact journals; involvement in renowned research projects or public health initiatives; participation in advisory groups or task forces for governments, health organisations or international bodies; and experience in developing guidelines or policies. Throughout the selection process, we sought disciplinary, sectoral and geographical diversity, with a focus on the WHO European Region.

Based on these criteria, 171 experts were invited to participate in the study, with 54 (31.58%) completing the first round, representing an almost even split between scholars (n=28) and RCCE-IM professionals (n=26). Of these, 34 experts (62.96%) participated in the second round. This exceeded the typical range of 8–20 participants in Delphi studies.^38^ The experts represented 27 countries and had an average of 16.98 years of professional experience (SD=8.98; see online supplemental appendix B). A large proportion of the panel reported expertise in risk communication (74.07%), public health (50%) and community engagement (37.03%), with 59.26% using AI daily or weekly in both professional and non-professional contexts. Of the RCCE-IM professionals, 57.7% indicated that their work already involves the use or development of AI. Most experts rated their knowledge of AI as basic (46.30%) to solid (40.74%).

### Patient and public involvement

It was not appropriate or possible to involve patients or the public in the design, or conduct, or reporting, or dissemination plans of our research.

## Results

### AI opportunities: enhancing risk communication and infodemic management

In the initial round of this Delphi study, experts identified 21 distinct opportunities for AI applications in RCCE-IM, which we organised into five overarching domains (see table 1). In addition to specific benefits for *risk communication* (eg, tailoring messages), *community engagement* (eg, virtual town hall meetings), and *infodemic management* (eg, identifying information voids), experts highlighted potential possibilities beyond RCCE-IM, including *operational management* (eg, resource allocation) and *evaluation and learning* (eg, intervention evaluation).

**Table 1:** Overview of opportunities, challenges, risks and actions

Opportunities of AI for RCCE-IM				
**Risk communication**	**Community engagement**	**Infodemic management**	**Operational management**	**Evaluation and learning**
Content generation and distribution Multilingual risk communication Simplifying messages Tailoring messages Good practice implementation Interactive dialogue	Community feedback management Interactive content and gamification Virtual town hall meetings	Social listening* Trend analysis* Identifying information voids False information detection False information correction Predictive analytics	Resource allocation Process optimisation Scenario simulation	Evidence synthesis Idea generation Intervention evaluation

Challenges and risks of AI for RCCE-IM					
**Algorithmic bias and misuse**	**Accessibility and inclusivity concerns**	**Privacy and transparency concerns**	**(Mis)information overload and deficit**	**Technological dependency and limitations**	**Organisational concerns**
Algorithmic bias Deepening disparities Malicious use	AI divide Data colonialism	Data privacy Algorithmic opacity Unclear accountability	Content verification Amplification bias Information overload Oversimplified communication Information deficit	Technological solutionism AI overreliance Provider dependency Lack of human values	Lack of resources Lack of regulations Job loss

Actions for the responsible use of AI for RCCE-IM				
**Policies, regulation and oversight**	**Resource allocation and implementation strategies**	**Improvement and feedback**	**Capacity building**	**Building public trust and education**
AI guidelines Continuous monitoring Oversight boards Alert systems Regulatory frameworks	Sufficient resources Infrastructure expansion Adapting technology Strategic implementation	Iterative improvement Human-in-the-loop User feedback Adaptive interventions	Societal collaboration Training programmes	Transparent communication Community involvement Educational campaigns

Detailed descriptions of the individual items are provided in online supplemental appendix C/D/E. Social Listening* and Trend Analysis* cut across all RCCE-IM components.

AI, artificial intelligence; RCCE-IM, risk communication, community engagement and infodemic management.

When asked to rank these opportunities, experts underscored that AI has the greatest potential to improve risk communication and infodemic management. According to the weighted ranking, content generation and distribution was identified as the most important application, with 44.12% of experts considering it one of the top three opportunities, followed by social listening (32.35%), tailoring messages (23.53%), multilingual risk communication (14.71%) and false information correction (14.71%) and detection (17.65%). In contrast, utilising AI to implement best practices or allocate resources (both mentioned by 2.94% of experts) ranked in the lower quartile and was therefore considered of marginal importance (see online supplemental appendix C for detailed descriptions of the opportunities and their rankings).

Leveraging AI to engage with communities was met with greater hesitation. One expert explained: ‘I am not convinced by AI’s potential in community engagement activities. If we hand over what should be very human processes to machines, we will not be in a position to show empathy to affected communities, and we will definitely not be building trust’ (E10).

### AI challenges and risks: mitigating algorithmic bias and privacy breaches

Experts associated 20 challenges and risks with the use of AI for RCCE-IM, which we grouped into six overarching domains (see table 1): *algorithmic bias and misuse* (eg, deepening disparities), *accessibility and inclusivity concerns* (eg, data colonialism), *privacy and transparency concerns* (eg, algorithmic opacity), (*mis)information overload and deficit* (eg, amplification bias), *technological dependency and limitations* (eg, technological solutionism) and *organisational concerns* (eg, lack of regulations). Notably, these challenges and risks were associated with all RCCE-IM components.

Among these challenges and risks, algorithmic bias was ranked as the most important, with 32.35% of experts considering it one of the top three concerns, followed by data privacy (32.35%), malicious use of AI (29.41%), content verification (29.41%), deepening disparities (17.65%) and AI overreliance (23.53%; see online supplemental appendix D). In contrast, the risks of oversimplified communication and provider dependency (each mentioned by 2.94% of experts) were seen as minor concerns and ranked in the lower quartile.

Building on concerns about technological solutionism and the tendency to underestimate AI’s limitations in RCCE-IM, experts further cautioned that ‘too many people are uncritical about these issues, and they want to embrace the technology as if it is a neutral actor―which it is not’ (E29). Additionally, several experts warned of AI ‘failing at the moment of emergency response in a crisis situation if we are too dependent on AI’ (E19).

Interestingly, not all experts agreed that AI lacks empathy. Some argued that AI is ‘capable of responding empathically to patients’, and noted that when under time pressure, healthcare providers might also ‘skip empathic communication altogether and focus solely on delivering factual information’ (E24).

### Future scenarios: increased proficiency but erosion of public trust

Experts’ predictions of how AI will transform RCCE-IM over the next 5 years revealed seven potential scenarios, with both *optimistic* and *pessimistic* outcomes. Optimistic scenarios included *increased proficiency* of AI, where technological advances improve the efficiency of AI-based RCCE-IM interventions and produce more accurate results with less need for human oversight. *Optimised workflows* envision AI reshaping organisational processes by automating routine tasks and freeing human resources for strategic work. Finally, *democratisation of information* suggests that AI will enable public health authorities to reach audiences more effectively by tailoring RCCE-IM interventions, promoting a more inclusive and participatory approach to managing health emergencies.

Pessimistic scenarios highlighted the *limited impact* of AI on RCCE-IM, as AI may struggle with complex tasks that require contextual understanding, empathy, ethical judgement and creative problem-solving, potentially resulting in generic interventions. Additionally, AI could contribute to a *surge of (mis)information* that contradicts and complicates the communication efforts of public health authorities. The *replacement of human tasks* by AI is another concern, as it may automate certain communicative roles, but without fully substituting human judgement. Finally, the use of AI for RCCE-IM could lead to an *erosion of public trust* by dehumanising sensitive communicative interventions alongside people’s fears of bias, opacity and privacy breaches, ultimately undermining confidence in health authorities.

**Figure 1:**
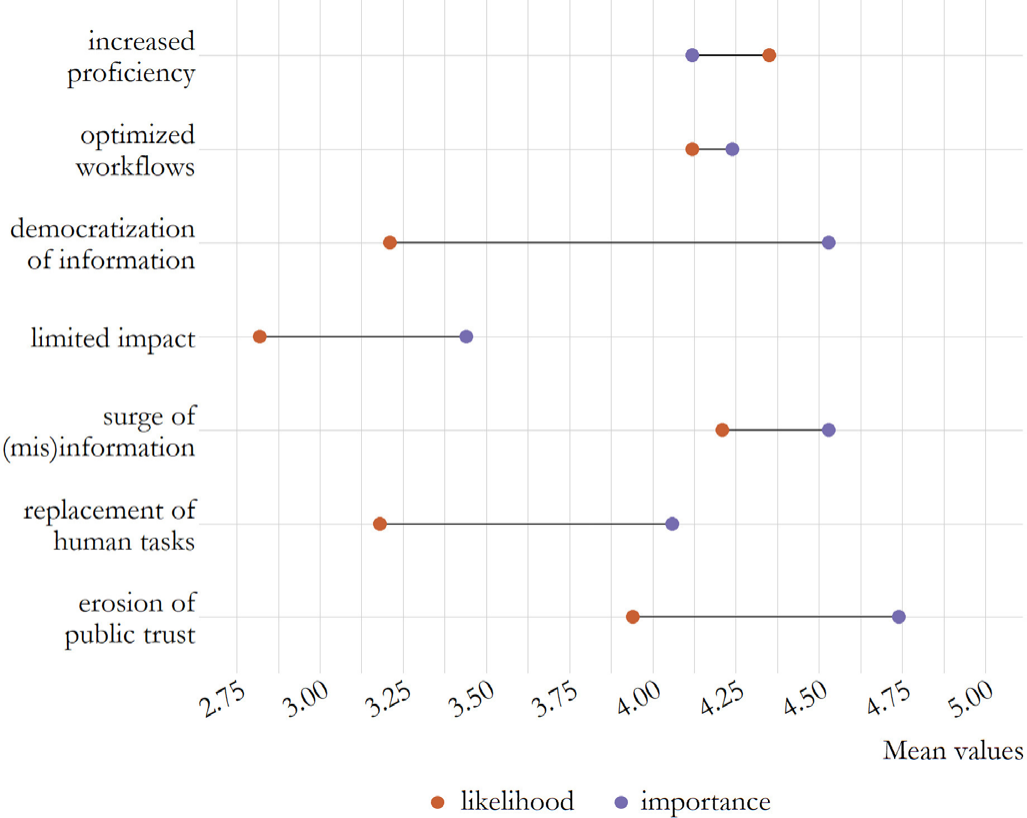
Likelihood and importance of future scenarios.

In the second wave, experts rated the likelihood and importance of these scenarios, with increased proficiency of AI identified as the most likely scenario, while the erosion of public trust due to AI was seen as the most urgent challenge to address. Notably, likelihood and importance did not always coincide, that is, scenarios considered highly important were not necessarily deemed highly likely (see figure 1): the democratisation of information, while considered somewhat likely (*M*=3.21, *SD*=1.20), was rated as very to extremely important (*M*=4.53, *SD*=0.90). Similar patterns were observed for the erosion of trust (likelihood: *M*=3.94, *SD*=1.07; importance: *M*=4.74, *SD*=0.57), the replacement of human tasks (likelihood: *M*=3.18, *SD*=1.42; importance: *M*=4.06, *SD*=1.25), and the limited impact of AI (likelihood: *M*=2.82, *SD*=1.36; importance: *M*=3.44, *SD*=1.40). Conversely, optimised workflows (likelihood: *M*=4.12, *SD*=0.95; importance: *M*=4.24, *SD*=1.16), increased proficiency (likelihood: *M*=4.35, *SD*=0.88; importance: *M*=4.12, *SD*=1.23) and a surge in (mis)information (likelihood: *M*=4.21, *SD*=1.09; importance: *M*=4.53, *SD*=1.05) were considered highly likely and important.

### Principles for responsible use of AI: equity, safety and transparency

**Figure 2:**
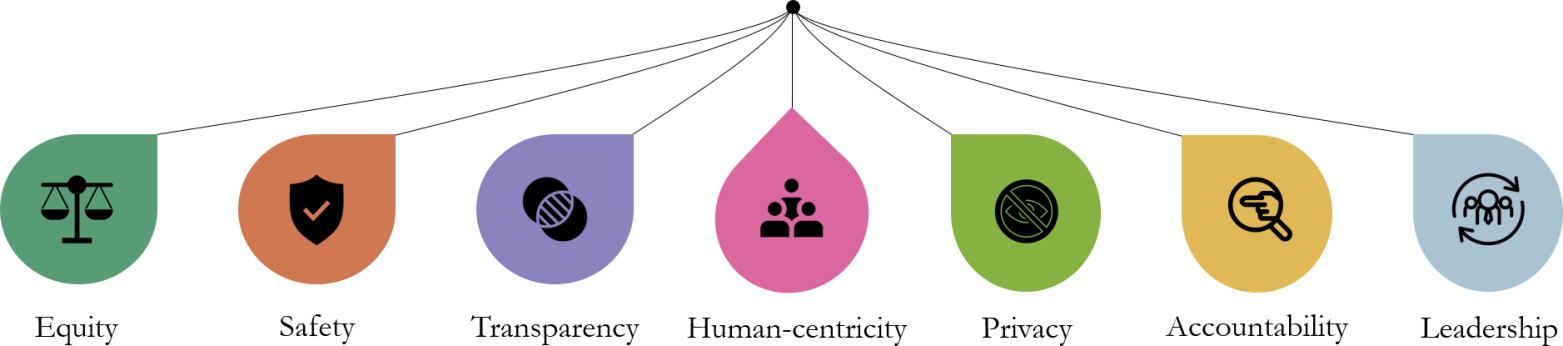
Principles for the responsible use of AI for RCCE-IM. AI, artificial intelligence; RCCE-IM, risk communication, community engagement and infodemic management.

Experts’ assessment of key factors that facilitate or impede the responsible use of AI for RCCE-IM interventions explicitly or implicitly pointed towards seven principles (see figure 2), which are presented below in order of their ranked importance (see online supplemental appendix E).

*Equity* seeks to ensure fairness, promote inclusivity, reduce discrimination and prevent algorithmic bias and stereotyping, particularly regarding sensitive information such as age, gender, ethnicity, socioeconomic status, sexual orientation, religion or medical records. Experts suggested that this can be achieved by using representative datasets and providing equitable access to information, which helps mitigate disparities affecting marginalised and vulnerable communities. 58.82% of the experts identified equity as one of the three most important principles.

*Safety* aims to promote the safe and robust use of AI by reducing the risk of malicious use, attacks or unauthorised access to sensitive health information. To achieve this, experts recommended designing and deploying AI systems that are resilient to disruptions, regularly stress testing these systems and implementing preventative measures to minimise unintended harm. 52.94% of the experts considered safety as one of the top three principles.

*Transparency* underscores the need to disclose the sources and origins of the data used, the analytical methods and criteria used to analyse the data and the rationale behind response strategies. Experts suggested that this can be achieved by designing AI systems that allow for human oversight and clearly communicating the ethical principles that govern their use. 58.82% of the experts considered transparency as one of the top three principles.

*Human-centricity* emphasises the prioritisation of human needs, norms and values throughout the development, deployment and governance of AI. According to the experts, this can be achieved by ensuring that AI-based RCCE-IM strategies are ethically sound, socially responsible and supportive of human rights. 41.18% of the experts considered human centricity as one of the top three principles.

*Privacy* focuses on protecting individual privacy. Experts suggested that this can be achieved through legislation that prevents infringement of privacy and by empowering individuals with control over their data, thereby enhancing public trust in the use of AI for RCCE-IM interventions. 35.29% of the experts considered privacy as one of the top three principles.

*Accountability* seeks to ensure accountability for the design, deployment and outcomes of AI. Experts recommended achieving this through robust accountability frameworks that define roles and responsibilities for developers, engineers, organisations, regulators and end users. Moreover, compliance mechanisms and consequences for non-compliance should be established. 35.29% of the experts considered accountability as one of the top three principles.

*Leadership* emphasises the importance of fostering a supportive and receptive organisational culture through responsible leadership. Experts suggested that this can be achieved by allocating resources for capacity building, ensuring robust infrastructure, fostering collaboration between public health authorities and societal stakeholders, implementing training programmes to increase AI literacy and developing policies that ensure responsible AI development, deployment and regulation. 17.65% of the experts considered leadership as one of the top three principles.

Overall, the three principles considered most important show that experts prioritise ethical over governance principles to ensure a responsible use of AI for RCCE-IM interventions. Equity and transparency (both 58.82%) fall into the top quartile, indicating particularly strong support among panellists. In contrast, privacy and accountability (both 35.29%) are positioned at the threshold of the lower quartile, while leadership (17.65%) clearly falls within it.

### Required actions for responsible use of AI: policies, regulation, oversight

Finally, experts proposed 18 actions needed to harness the opportunities, address the challenges and mitigate the risks presented by AI for RCCE-IM. These actions were grouped into five overarching domains (see table 1): *policies, regulation and oversight* (eg, AI guidelines), *resource allocation and implementation strategies* (eg, infrastructure expansion), *improvement and feedback* (eg, human-in-the-loop), *capacity building* (eg, societal collaboration), and *building public trust and education* (eg, community involvement).

According to the experts, regulatory frameworks (one of the top three actions for 50% of the panel), continuous monitoring (44.12%) and AI guidelines (29.41%) are the most critical for the responsible use of AI for RCCE-IM (see online supplemental appendix F). Additionally, public health authorities should build on the adoption of human-in-the-loop systems (26.47%), enhance societal collaboration (23.53%) and ensure transparent communication (11.76%). In contrast, measures such as expanding existing infrastructure (5.88%) or adapting interventions to recent AI developments (2.94%) were ranked in the lower quartile and considered less important. One expert also warned that the option of ‘opting out of AI’ (E22) needs to be considered.

## Discussion

Although AI is poised to reshape multiple health domains, scholarship has focused primarily on public health, medicine, clinical care, medical research and education,^14^,^18^ leaving scholars and public health professionals with an inadequate understanding of the technology’s larger transformative power on public health practices, including RCCE-IM interventions to manage health emergencies. This study is the first international, multidisciplinary and transdisciplinary effort to address this gap, employing a modified Delphi study to assess the opportunities, challenges and risks associated with AI, anticipate future scenarios and identify key principles and actions to facilitate the responsible use of AI for RCCE-IM.

According to this study’s expert panel, the greatest *opportunities* of AI lie in risk communication and infodemic management, while its application in community engagement is met with greater hesitation due to its lack of emotional, cognitive and motivational empathy―an issue that remains the subject of ongoing debate about AI’s capacity for empathy.^53^,^55^ Experts’ optimism regarding AI’s potential for health emergencies, particularly in content generation and distribution, is coupled with concerns about *challenges and risks*, with algorithmic bias, privacy issues and the malicious use of AI being the greatest risks that require urgent attention. These concerns mirror threats discussed in other fields^2656^,^59^ and echo the *principles* and *actions* proposed for the responsible use of AI in public health crises. According to these principles, the responsible application of AI should prioritise equity and transparency,^11^ supported by the implementation of regulatory frameworks, the development of AI guidelines and ongoing monitoring. Finally, the experts’ anticipation of *transformative scenarios* underscores the need for public health authorities to proactively leverage AI to democratise information, while addressing the risk that opaque use of the technology could lead to an erosion of trust in health organisations.

### Limitations

These results should be considered in light of this study’s limitations. First, the Delphi method has been criticised for potential ‘homophily bias’,^60^ which we attempted to minimise by increasing the diversity of participants. Second, the method relies on the continued participation of panellists over multiple rounds, which is why Delphi studies often suffer from declining response rates^38^; to account for this, we modified the design of this Delphi study and recruited a larger panel of experts that would be more resilient to such declines. Third, since this Delphi study consisted of two survey rounds, combining open-ended and ranking questions without repeating the exact same questions multiple times, response stability could not be measured. To partially address this limitation, experts were asked to provide feedback on the results, allowing them to change their assessment in light of their peers’ responses; however, they did not express significant disagreement or a desire to modify their initial judgment. Fourth, since experts identified a total of 66 distinct opportunities, challenges, principles and actions, the subsequent weighted ranking was deliberately limited to the three most important items. Ranking all items on Likert scales would have been too granular and likely increased dropout rates. However, this approach also meant that panellists were unable to assess the importance of the remaining items. Fifth, this study was conducted in English, which may have inadvertently excluded the perspectives of experts who are not proficient in English.

### Conclusion

This study highlights the challenge of responsibly harnessing the opportunities of AI to manage public health emergencies amidst significant risks. This is particularly evident in AI’s double-edged nature: while it can, for instance, improve targeted risk communication and thus promote inclusivity, it also carries the risk of exacerbating existing health disparities and inequalities, especially in rural, underprivileged communities. Navigating this duality responsibly requires societal collaboration. Hence, future efforts will need to determine the forms of collaboration needed, identify relevant stakeholders and develop measures that are internationally relevant^61^ while being sensitive to specific cultures, regions, organisations and technologies. Guideline adaptation frameworks^62 63^ can facilitate this process by offering a structured approach to ensure transparency and methodological rigour. This Delphi study is intended to serve as a first step towards this goal, providing evidence-based and actionable guidance to promote the responsible use of AI for RCCE-IM in public health emergencies.

## Supplementary material

10.1136/bmjgh-2024-018545 online supplemental file 1
