# Supplementary material for: Responsible artificial intelligence in public health: a Delphi study on risk communication, community engagement and infodemic management
Source: BMJ Glob Health. 2025 May 23;10(5):e018545. doi: 10.1136/bmjgh-2024-018545 (PMC12104889; doi:10.1136/bmjgh-2024-018545)
Supplement: online supplemental file 1 [file bmjgh-10-5-s001.pdf]

Supplemental Appendix for

Daniela Mahl, Mike S. Schäfer, Stefan Adrian Voinea, Keyrellous Adib, Ben Duncan, Cristiana Salvi, David Novillo-Ortiz

Responsible Artificial Intelligence in Public Health: A Delphi Study on Risk Communication, Community Engagement, and Infodemic Management

published in *BMJ Global Health*

ABSTRACT

Introduction Artificial intelligence (AI) holds the potential to fundamentally transform how public health authorities utilize risk communication, community engagement, and infodemic management (RCCE-IM) to prepare for, manage, and mitigate public health emergencies. As research on this crucial transformation remains limited, we conducted a modified Delphi study on the impact of AI on RCCE-IM.

Methods In two successive surveys, 54 experts—scholars with expertise in public health, digital health, health communication, risk communication, and AI, as well as RCCE-IM professionals—from 27 countries assessed opportunities, challenges, and risks of AI, anticipated future scenarios, and identified principles and actions to facilitate the responsible use of AI. The first Delphi round followed an open, exploratory approach, while the second sought to prioritize and rank key findings from the initial phase. Qualitative thematic analysis and statistical methods were applied to evaluate responses.

Results According to the expert panel, AI could be highly beneficial, particularly for risk communication (e.g., tailoring messages) and infodemic management (e.g., social listening), while its utility for fostering community engagement was viewed more critically. Challenges and risks affect all three components of RCCE-IM equally, with algorithmic bias and privacy breaches being of particular concern. Panelists anticipated both optimistic (e.g., democratization of information) and pessimistic (e.g., erosion of public trust) future scenarios. They identified seven principles for the responsible use of AI for public health practices, with equity and transparency being the most important. Prioritized actions ranged from regulatory measures, resource allocation, and feedback loops to capacity building, public trust initiatives, and educational training.

Conclusion To responsibly navigate the multifaceted opportunities, challenges, and risks of AI for RCCE-IM in public health emergencies, clear guiding principles, ongoing critical evaluation and training, as well as societal collaboration across countries are needed.

Content

Appendix A: Delphi design 3
Appendix B: Delphi expert panel 6
Appendix C: AI opportunities for RCCE-IM 8
Appendix D: AI challenges and risks for RCCE-IM 10
Appendix E: Principles for responsible AI use for RCCE-IM 12
Appendix F: Required actions for responsible AI use for RCCE-IM 13

Appendix A: Delphi design

Supplementary Table 1. Survey questionnaires

	Dimension	Question
**1st round**	**Online survey** (*initial assessment*)	
	**Professional background**	My professional area of expertise and practice lies in the field(s) of …<br>1 = public health<br>2 = digital health<br>3 = risk communication<br>4 = community engagement<br>5 = infodemic management<br>6 = artificial intelligence<br>7 = other (open-field text box)<br><br>Which of the following describes your work best?<br>1 = I am a researcher, working either in academia or in a non-academic context (e.g., a civil society organization)<br>2 = I am a practitioner, working for a public health authority<br>3 = other (open-field text box)<br><br>Please specify your professional background:<br>1 = Professional role or position (open-field text box)<br>2 = Name of institution/organization (open-field text box)<br>3 = Country in which you mainly work (open-field text box)<br>4 = Years of professional experience in the field (open-field text box)
	**Experience in using AI**	How often do you use AI tools, both professionally and in other contexts?<br>1 = At least once a day<br>2 = At least once a week<br>3 = At least once a month<br>4 = Less often<br>5 = Never

	Does your professional practice involve the use or development of AI? (only for RCCE-IM professionals)<br>1 = Yes<br>2 = No
**Self-assessed knowledge of AI**	How would you rate your knowledge about AI?<br>1 = Advanced knowledge (I know about possible applications of AI as well as opportunities, challenges, and risks tailored to specific contexts and different subtypes of AI)<br>2 = Solid knowledge (I know about possible applications of AI as well as opportunities, challenges, and risks in general, but not tailored to specific contexts nor to different subtypes of AI)<br>3 = Basic knowledge (I have some knowledge of potential applications of AI)<br>4 = No or limited knowledge (I don't know or have only heard of possible applications of AI)
**AI opportunities**	What opportunities does AI, and especially generative AI, present for the risk communication, community engagement, and infodemic management of public health authorities?<br>Please indicate if any of the identified opportunities apply only to risk communication, only to community engagement, or only to infodemic management. If possible, please briefly illustrate identified opportunities with examples. (open-field text box)
**AI challenges and risks**	What challenges and risks does AI, and especially generative AI, present for the risk communication, community engagement, and infodemic management of public health authorities?<br>Please indicate if any of the identified risks and challenges apply only to risk communication, only to community engagement, or only to infodemic management. If possible, please briefly illustrate identified challenges and risks with examples. (open-field text box)
**Future AI scenarios**	How will AI, and especially generative AI, transform the risk communication, community engagement, and infodemic management of public health authorities in the next 5 years?<br>Please indicate if any of the discussed transformative scenarios apply only to risk communication, only to community engagement, or only to infodemic management. If possible, please briefly illustrate discussed transformations with examples. (open-field text box)
**Principles for responsible AI use**	What are the key factors that facilitate or prevent the responsible use of AI, and especially generative AI, for the risk communication, community engagement, and infodemic management of public health authorities?<br>Please indicate if any of the identified facilitators and inhibitors apply only to risk communication, only to community engagement, or only to infodemic management. If possible, please briefly illustrate identified facilitators and inhibitors with examples. (open-field text box)
**Required actions for AI implementation**	What actions are needed to harness the opportunities, address the challenges, and mitigate the risks of AI, especially generative AI, for the risk communication, community engagement, and infodemic management of public health authorities?<br>Please indicate if any of the discussed actions apply only to risk communication, only to community engagement, or only to infodemic management. If possible, please briefly illustrate discussed actions with examples. (open-field text box)

2nd round	Online survey<br>(*ranking round*)	
	**AI opportunities**	Of all the opportunities above (highlighted in blue), which three are the most important for public health authorities' RCCE-IM? (open-field text box)<br><br>Are there other opportunities that are not covered in the above list, or do you disagree with any of the opportunities listed? (open-field text box)
	**AI challenges and risks**	Of all the challenges and risks above (highlighted in blue), which three are the most important for public health authorities' RCCE-IM? (open-field text box)<br><br>Are there other challenges and risks that are not covered in the above list, or do you disagree with any of the challenges and risks listed? (open-field text box)
	**Future AI scenarios**	Over the next 5 years, how likely do you think each of these scenarios is?<br>(1 = "not at all likely" to 5 = "very likely", including "don't know" option)<br><br>Over the next 5 years, how important is it for public health authorities to address these scenarios?<br>(1 = "not at all important" to 5 = "very important", including "don't know" option)<br><br>Are there other scenarios that are not covered in the above list, or do you disagree with any of the scenarios listed? (open-field text box)
	**Principles for responsible AI use**	Of all the principles above (highlighted in blue), which three are the most important for public health authorities' RCCE-IM? (open-field text box)<br><br>Are there other principles that are not covered in the above list, or do you disagree with any of the principles listed? (open-field text box)
	**Required actions for AI implementation**	Of all the actions above (highlighted in blue), which three are the most important for public health authorities' RCCE-IM? (open-field text box)<br><br>Are there other actions needed that are not covered in the above list, or do you disagree with any of the actions listed? (open-field text box)

Appendix B: Delphi expert panel

Scholarly experts included professors, senior researchers, physicians, chief medical officers, as well as scientific directors and coordinators affiliated with universities, research centers, national ministries and institutes of public health, and international public health organizations. *RCCE-IM professionals* included risk communication advisors, consultants, officers, project coordinators, directors, and managers employed by national, international, and intergovernmental public health institutions and ministries, as well as general medical journals. A large proportion of the expert panel reported expertise in risk communication (74.07%), public health (50%), and community engagement (37.03%) (see Supplementary Figure 1), with 31.48% using AI daily and 27.78% weekly in both professional and non-professional contexts (see Supplementary Figure 2). Most experts rated their knowledge of AI as basic (46.30%) to solid (40.74%) (see Supplementary Figure 3).

Supplementary Figure 1. Experts' area of specialization

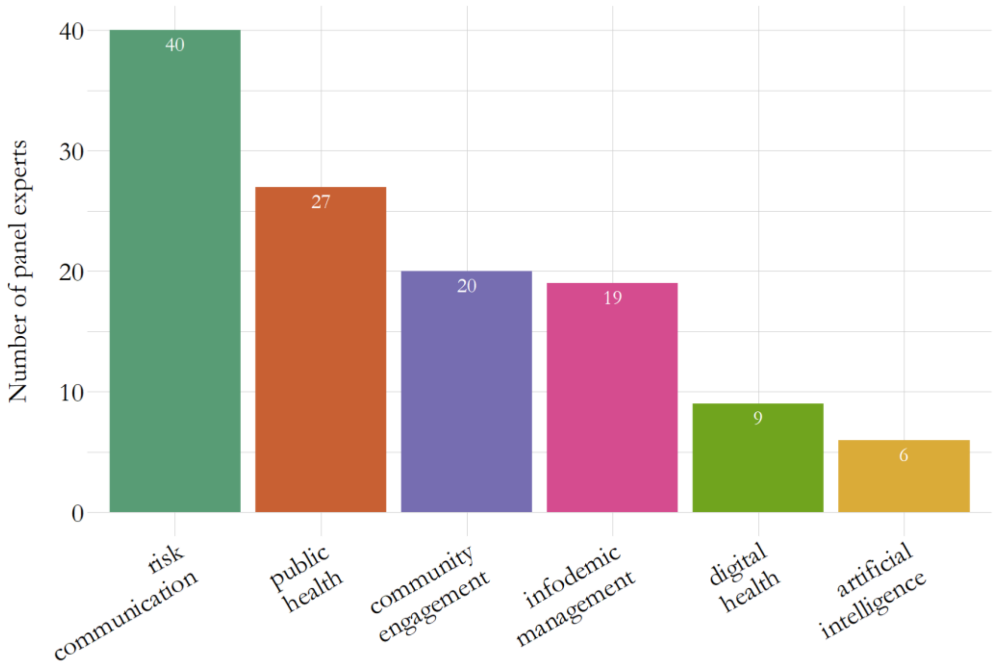

Note. N_{all} = 54 experts.

Supplementary Figure 2. Experts' experience in using AI

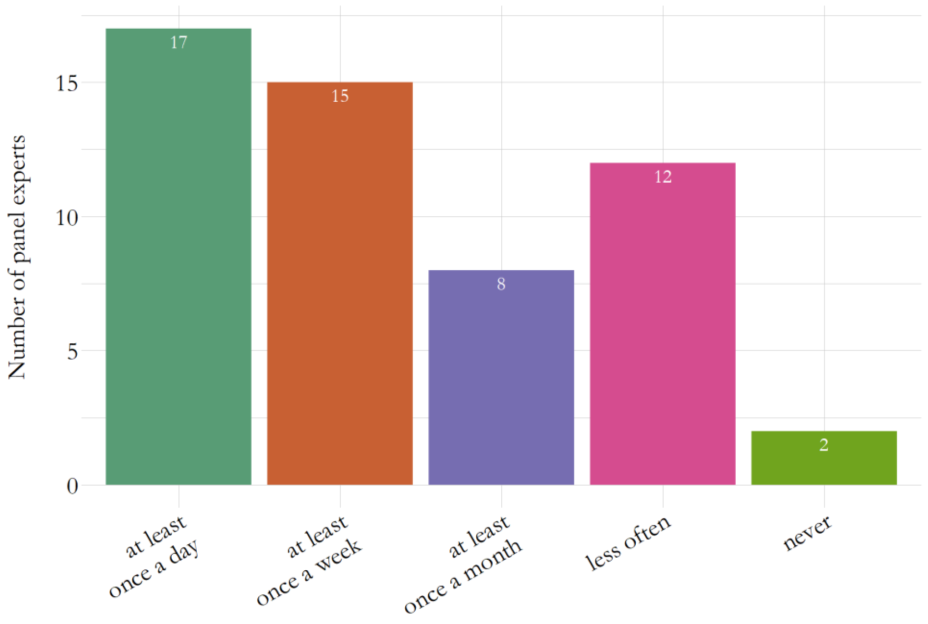

Note. N_{all} = 54 experts.

Supplementary Figure 3. Experts' self-assessed knowledge of AI

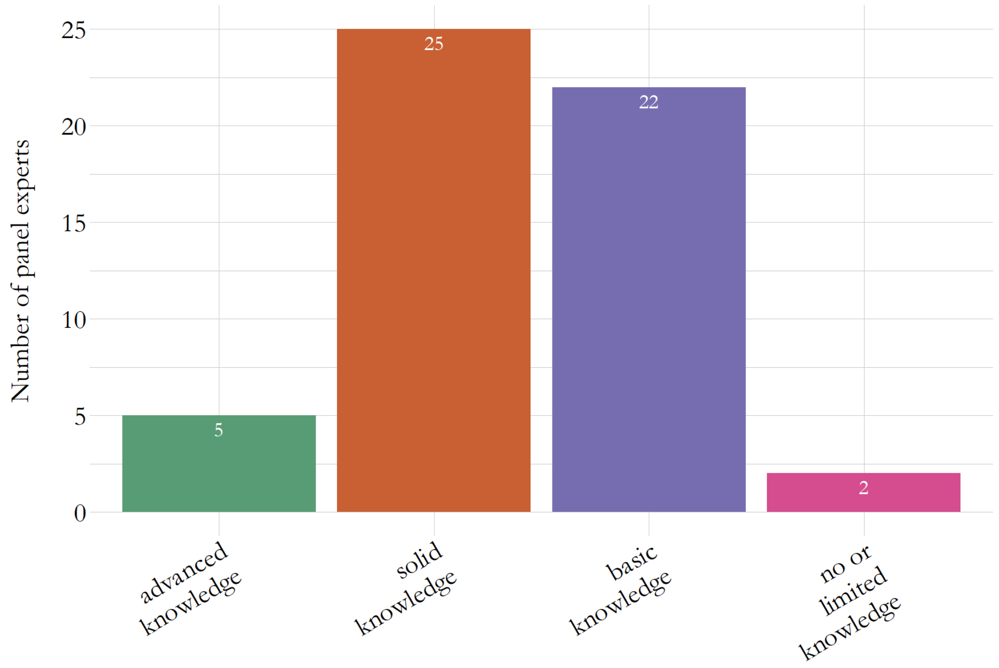

Note. N_{all} = 54 experts.

Appendix C: AI opportunities for RCCE-IM

Supplementary Table 2. Opportunities of AI for RCCE-IM

Opportunities	Short description
Risk communication	
Content generation and distribution	AI can rapidly and efficiently create health messages in textual, visual, or other forms, and support multi-channel messaging to improve emergency response.
Multilingual risk communication	AI can generate multilingual, culture-sensitive content, making health information accessible to diverse populations and risk communication more inclusive.
Simplifying messages	AI can translate scientific information into accessible language or simplify website navigation, improving responsiveness and accessibility for users.
Tailoring messages	AI can help test and personalize health messages that resonate more effectively with different populations, increasing the likelihood of compliance with health recommendations.
Good practice implementation	AI can facilitate the implementation of good practices in risk communication, ensuring consistent and effective dissemination of critical information.
Interactive dialogue	AI can simulate human-like interactions through chatbots and virtual assistants, actively engaging with audiences and addressing concerns quickly.
Community engagement	
Community feedback management	AI-powered chatbots can triage inquiries and deliver accurate information and guidance promptly, especially during health emergencies when timely responses are critical.
Interactive content and gamification	AI can develop interactive and engaging educational tools, such as quizzes and games, to educate communities about health risks and preventive measures.
Virtual town hall meetings	AI can facilitate virtual community workshops to disseminate health information and promote interactive learning experiences for the public.
Infodemic management	
Social listening*	AI can monitor social media and other online platforms to gauge public sentiment, helping public health authorities understand public concerns and respond appropriately.
Trend analysis*	AI can identify general trends in public health data, helping authorities understand and anticipate public health needs.
Identifying information voids	AI can analyze large datasets and identify gaps in available information, highlighting areas where additional information is needed.
False information detection	AI can detect false information by leveraging real-time data analysis to identify patterns indicative of false or misleading information.
False information correction	AI can fact-check and correct false information, delivering customized correction and debunking material across multiple channels.
Predictive analytics	AI can predict which false information trends are likely to spread, enabling preemptive action to counter false narratives before they gain traction.
Operational management	
Resource allocation	AI can help optimize the allocation of resources such as infrastructure and personnel by analyzing data and predicting needs based on emerging trend.
Process optimization	AI can streamline processes by automating routine and repetitive tasks, thereby optimizing workflow efficiency and reducing the workload of public health professionals.

Scenario simulation	AI can simulate scenarios by creating realistic, data-driven models of potential emergencies, allowing public health professionals to practice and refine their responses in controlled environments.
Evaluation and learning	
Evidence synthesis	AI can aggregate scientific evidence by rapidly synthesizing data from multiple studies and sources, enabling the development of robust, evidence-based interventions.
Idea generation	AI can facilitate collaborative brainstorming and evaluate new ideas across diverse scenarios, helping to refine strategies for more effective outcomes.
Intervention evaluation	AI can evaluate outcomes of interventions to support evidence-based decision-making and refine strategies as needed.

*Note. Social Listening** and *Trend Analysis** cut across all RCCE-IM components.

Supplementary Table 3. Ranking of AI opportunities for RCCE-IM

Opportunities	Top1<br>N (%)	Top2<br>N (%)	Top3<br>N (%)	Top1-3 Sum<br>N (%)	Rank
Content generation and distribution	12 (35.29)	0 (0.00)	3 (8.82)	15 (44.12)	39
Social listening	3 (8.82)	6 (17.65)	2 (5.88)	11 (32.35)	23
Tailoring messages	2 (5.88)	3 (8.82)	3 (8.82)	8 (23.53)	15
Multilingual risk communication	2 (5.88)	2 (5.88)	1 (2.94)	5 (14.71)	11
False information correction	2 (5.88)	2 (5.88)	1 (2.94)	5 (14.71)	11
False information detection	1 (2.94)	3 (8.82)	2 (5.88)	6 (17.65)	11
Predictive analytics	2 (5.88)	1 (2.94)	2 (5.88)	5 (14.71)	10
Scenario simulation	0 (0.00)	3 (8.82)	4 (11.76)	7 (20.59)	10
Interactive dialogue	2 (5.88)	1 (2.94)	1 (2.94)	4 (11.76)	9
Evidence synthesis	2 (5.88)	1 (2.94)	1 (2.94)	4 (11.76)	9
Trend analysis	1 (2.94)	2 (5.88)	1 (2.94)	4 (11.76)	8
Community feedback management	0 (0.00)	3 (8.82)	2 (5.88)	5 (14.71)	8
Idea generation	1 (2.94)	1 (2.94)	1 (2.94)	3 (8.82)	6
Process optimization	0 (0.00)	1 (2.94)	4 (11.76)	5 (14.71)	6
Identifying information voids	1 (2.94)	0 (0.00)	2 (5.88)	3 (8.82)	5
Intervention evaluation	0 (0.00)	1 (2.94)	3 (8.82)	4 (11.76)	5
Simplifying messages	1 (2.94)	0 (0.00)	1 (2.94)	2 (5.88)	4
Good practice implementation	1 (2.94)	0 (0.00)	0 (0.00)	1 (2.94)	3
Resource allocation	1 (2.94)	0 (0.00)	0 (0.00)	1 (2.94)	3
Interactive content and gamification	0 (0.00)	1 (2.94)	0 (0.00)	1 (2.94)	2
Virtual town hall meetings	0 (0.00)	0 (0.00)	0 (0.00)	0 (0.00)	0

Note. N_{all} = 34 experts; rank is calculated as the sum of Top1x3 + Top2x2 + Top3.

Appendix D: AI challenges and risks for RCCE-IM

Supplementary Table 4. Challenges and risks of AI for RCCE-IM

Challenges and risks	Short description
Algorithmic bias and misuse	
Algorithmic bias	AI may perpetuate biases, stereotypes, and discrimination present in training data, potentially leading to distorted interventions that could disproportionately affect marginalized, vulnerable communities.
Deepening disparities	AI may exacerbate public health disparities by disproportionately allocating attention and resources based on biased data, further marginalizing communities that may be underrepresented in the training data.
Malicious use	AI may be weaponized for illegitimate disease surveillance or unintentionally misapplied, which can undermine public trust and harm public health initiatives.
Accessibility and inclusivity concerns	
AI divide	The access to and literacy skills needed to use AI may be unevenly distributed, leaving underserved populations with less effective RCCE-IM interventions.
Data colonialism	AI may lead to a divide between those who develop AI and control the training data, and those who use AI and provide the data but have little control over its use.
Privacy and transparency concerns	
Data privacy	AI often relies on large datasets, raising concerns about the privacy of sensitive health information and the potential misuse of personal data.
Algorithmic opacity	The inner workings of AI systems remain often opaque, making it difficult to understand how decisions are made.
Unclear accountability	The opacity of AI systems may complicate identifying and holding responsible parties accountable for false information or ineffective risk communication strategies.
(Mis)information overload and deficit	
Content verification	Verifying the accuracy and credibility of AI-generated content poses a challenge for ensuring reliable risk communication.
Amplification bias	AI may amplify inaccurate, misleading, or outdated information by hallucinating and producing convincing but false content, exacerbating infodemic challenges.
Information overload	AI can contribute to information overload by generating vast amounts of data, making it difficult for public health authorities to cut through the noise with official information.
Oversimplified communication	Oversimplification of information can fail to adequately convey the complexity of science, potentially leading to ineffective understanding of recommendations.
Information deficit	Overly AI-driven tailored risk communications may omit relevant information, potentially failing to provide a comprehensive view of health risks or prevention strategies.
Technological dependency and limitations	
Technological solutionism	Overestimating the benefits and dismissing the challenges and risks of AI can lead to underestimating the importance of non-digital forms of RCCE-IM.

AI overreliance	Too much reliance on AI can diminish human creativity, critical thinking, and intuition, which are essential for making nuanced decisions in complex public health situations, such as emergencies.
Provider dependency	Dependency on commercial, corporate providers can result in a lack of tools designed specifically for the use by public health authorities, hindering their ability to communicate effectively.
Lack of human values	AI may lack the empathy and contextual understanding needed to understand the diverse cultural and social factors that influence public health outcomes, potentially leading to detached responses to community needs and an erosion of trust.
Organizational concerns	
Lack of resources	Limited investment in developing and deploying AI can hamper public health authorities' ability to leverage advanced technologies for innovative RCCE-IM interventions, impeding their capacity to effectively address complex public health challenges.
Lack of regulations	The lack of regulatory guidelines and standards for the use of AI can lead to inconsistent practices, ethical dilemmas, and potential misuse, potentially undermining public trust.
Job loss	The increased reliance and improved performance of AI may lead to job replacement as tasks traditionally performed by humans are automated.

Supplementary Table 5. Ranking of AI challenges and risks for RCCE-IM

Challenges and risks	Top1 N (%)	Top2 N (%)	Top3 N (%)	Top1-3 Sum N (%)	Rank
Algorithmic bias	9 (26.47)	0 (0.00)	2 (5.88)	11 (32.35)	29
Data privacy	6 (17.65)	4 (11.76)	1 (2.94)	11 (32.35)	27
Malicious use	2 (5.88)	4 (11.76)	4 (11.76)	10 (29.41)	18
Content verification	2 (5.88)	4 (11.76)	4 (11.76)	10 (29.41)	18
Deepening disparities	4 (11.76)	0 (0.00)	2 (5.88)	6 (17.65)	14
AI overreliance	1 (2.94)	4 (11.76)	3 (8.82)	8 (23.53)	14
Lack of regulations	2 (5.88)	3 (8.82)	1 (2.94)	6 (17.65)	13
Lack of human values	2 (5.88)	2 (5.88)	2 (5.88)	6 (17.65)	12
AI divide	2 (5.88)	2 (5.88)	1 (2.94)	5 (14.71)	11
Technological solutionism	1 (2.94)	1 (2.94)	4 (11.76)	6 (17.65)	9
Unclear accountability	0 (0.00)	4 (11.76)	1 (2.94)	5 (14.71)	9
Algorithmic opacity	2 (5.88)	0 (0.00)	0 (0.00)	2 (5.88)	6
Lack of resources	1 (2.94)	0 (0.00)	2 (5.88)	3 (8.82)	5
Information overload	0 (0.00)	2 (5.88)	1 (2.94)	3 (8.82)	5
Amplification bias	0 (0.00)	1 (2.94)	1 (2.94)	2 (5.88)	3
Data colonialism	0 (0.00)	1 (2.94)	0 (0.00)	1 (2.94)	2
Information deficit	0 (0.00)	1 (2.94)	0 (0.00)	1 (2.94)	2
Job loss	0 (0.00)	0 (0.00)	2 (5.88)	2 (5.88)	2
Oversimplified communication	0 (0.00)	0 (0.00)	1 (2.94)	1 (2.94)	1
Provider dependency	0 (0.00)	0 (0.00)	1 (2.94)	1 (2.94)	1

Note. N_{all} = 34 experts; rank is calculated as the sum of Top1x3 + Top2x2 + Top3.

Appendix E: Principles for responsible AI use for RCCE-IM

Supplementary Table 6. Ranking of principles for responsible AI use for RCCE-IM

Principles	Top1 N (%)	Top2 N (%)	Top3 N (%)	Top1-3 Sum N (%)	Rank
Equity	10 (29.41)	7 (20.59)	3 (8.82)	20 (58.82)	47
Safety	5 (14.71)	8 (23.53)	5 (14.71)	18 (52.94)	36
Transparency	4 (11.76)	7 (20.59)	9 (26.47)	20 (58.82)	35
Human-centricity	7 (20.59)	4 (11.76)	3 (8.82)	14 (41.18)	32
Privacy	4 (11.76)	3 (8.82)	5 (14.71)	12 (35.29)	23
Accountability	2 (5.88)	3 (8.82)	7 (20.59)	12 (35.29)	19
Leadership	2 (5.88)	2 (5.88)	2 (5.88)	6 (17.65)	12

Note. N_{all} = 34 experts; rank is calculated as the sum of Top1x3 + Top2x2 + Top3.

Appendix F: Required actions for responsible AI use for RCCE-IM

Supplementary Table 7. Required actions for responsible use of AI for RCCE-IM

Required actions	Short description
Policies, regulation, and oversight	
AI guidelines	Developing and implementing guidelines to ensure the responsible use of AI for RCCE-IM interventions, detailing clear principles and standards to ensure effective dissemination of information and to mitigate risks associated with bias, privacy breaches, or false information.
Continuous monitoring	Establishing mechanisms to continually evaluate and update implemented AI guidelines to adapt to evolving technologies and address emerging ethical or operational challenges.
Oversight boards	Conducting regular audits to ensure that the development, deployment, governance, and AI-generated outputs comply with and maintain established principles set forth in AI guidelines.
Alert systems	Developing and implementing alert systems for malicious uses of AI to quickly identify and mitigate threats.
Regulatory frameworks	Developing and implementing (trans)national regulatory frameworks to ensure the responsible use of AI for public health and beyond, such as the EU Artificial Intelligence Act, including ongoing evaluation and possible adaptation.
Resource allocation and implementation strategies	
Sufficient resources	Allocating sufficient funding and resources to develop, implement, and maintain AI-driven initiatives to enable robust, scalable, and sustainable solutions to public health challenges.
Infrastructure expansion	Ensuring that the necessary technological infrastructure is in place to effectively use AI for RCCE-IM interventions and to reach all target communities, including remote and underserved areas.
Adapting technology	Supporting the continuous improvement, customization, and integration of AI systems specific to RCCE-IM interventions to ensure that these systems remain responsive to evolving public health needs and challenges in emergencies.
Strategic implementation	Developing a strategic plan for phased implementation of AI for RCCE-IM interventions to ensure a structured, efficient, and scalable approach that maximizes benefits while mitigating challenges and risks.
Improvement and feedback	
Iterative improvement	Adopting an iterative approach to continuously refine and enhance implemented AI systems to ensure they adapt to changing needs and incorporate feedback.
Human-in-the-loop	Establishing robust human-in-the-loop systems to verify the accuracy of AI-generated content, minimize bias, protect sensitive health information, and ensure transparency of AI operations.
User feedback	Implementing regular feedback from the community stakeholders and public health authorities to ensure that AI-based intervention strategies are responsive to real-world needs and challenges.
Adaptive interventions	Updating RCCE-IM interventions to ensure they remain adaptable, relevant, and effective to keep pace with AI developments.
Capacity building	

Societal collaboration	Fostering responsible partnerships with technology companies, global corporations, policymakers, researchers, and civil society to stay abreast of the latest advances, best practices, and community-specific needs.
Training programs	Investing in capacity-building activities for RCCE-IM practitioners to equip them with the knowledge and skills necessary to harness the potential of AI to improve the reach, impact, and accuracy of their efforts in health emergencies.
Building public trust and education	
Transparent communication	Clearly communicating the benefits and limitations of AI in public health initiatives to manage expectations and build trust.
Community involvement	Engaging community leaders and influencers to build trust and increase the effectiveness of RCCE-IM interventions.
Educational campaigns	Conducting public campaigns to improve AI literacy so that individuals have the knowledge and skills necessary to critically evaluate AI-driven information.

Supplementary Table 8. Ranking of required actions for responsible AI use for RCCE-IM

Required actions	Top1 N (%)	Top2 N (%)	Top3 N (%)	Top1-3 Sum N (%)	Rank
Regulatory frameworks	10 (29.41)	5 (14.71)	2 (5.88)	17 (50.00)	42
Continuous monitoring	6 (17.65)	4 (11.76)	5 (14.71)	15 (44.12)	31
AI guidelines	4 (11.76)	5 (14.71)	1 (2.94)	10 (29.41)	23
Human-in-the-loop	5 (14.71)	2 (5.88)	2 (5.88)	9 (26.47)	21
Societal collaboration	2 (5.88)	3 (8.82)	3 (8.82)	8 (23.53)	15
Transparent communication	2 (5.88)	1 (2.94)	1 (2.94)	4 (11.76)	9
Strategic implementation	1 (2.94)	3 (8.82)	0 (0.00)	4 (11.76)	9
Training programs	0 (0.00)	2 (5.88)	4 (11.76)	6 (17.65)	8
Educational campaigns	2 (5.88)	0 (0.00)	1 (2.94)	3 (8.82)	7
Sufficient resources	0 (0.00)	2 (5.88)	3 (8.82)	5 (14.71)	7
Iterative improvement	1 (2.94)	1 (2.94)	1 (2.94)	3 (8.82)	6
Adapting technology	0 (0.00)	3 (8.82)	0 (0.00)	3 (8.82)	6
Community involvement	0 (0.00)	0 (0.00)	5 (14.71)	5 (14.71)	5
Alert systems	1 (2.94)	0 (0.00)	1 (2.94)	2 (5.88)	4
User feedback	0 (0.00)	1 (2.94)	2 (5.88)	3 (8.82)	4
Oversight boards	0 (0.00)	1 (2.94)	1 (2.94)	2 (5.88)	3
Infrastructure expansion	0 (0.00)	1 (2.94)	1 (2.94)	2 (5.88)	3
Adaptive interventions	0 (0.00)	0 (0.00)	1 (2.94)	1 (2.94)	1

Note. N_{all} = 34 experts; rank is calculated as the sum of Top1x3 + Top2x2 + Top3.
